# Comparison of Conventional and Femtosecond Laser-Assisted Cataract Surgery Regarding Macula Behavior and Thickness

**DOI:** 10.3390/medicina59040639

**Published:** 2023-03-23

**Authors:** Filip Slezak, Gabriele Thumann, Martina Kropp, Zeljka Cvejic, Eline Elodie Barbara De Clerck, Giorgio Enrico Bravetti, Ivo Guber, Bojan Pajic

**Affiliations:** 1 Eye Clinic ORASIS, Swiss Eye Research Foundation, 5734 Reinach, Switzerland; 2 Division of Ophthalmology, Department of Clinical Neurosciences, Geneva University Hospitals, 1205 Geneva, Switzerland; 3 Experimental Ophthalmology, University of Geneva, 1205 Geneva, Switzerland; 4 Faculty of Sciences, Department of Physics, University of Novi Sad, Trg Dositeja Obradovica 4, 21000 Novi Sad, Serbia; 5 Faculty of Medicine of the Military Medical Academy, University of Defense, 11000 Belgrade, Serbia

**Keywords:** retinal thickness, femtosecond laser-assisted cataract surgery (FLACS), conventional cataract surgery

## Abstract

*Background*: The aim of the study was to compare macular thickness behavior and clinical outcomes after femtosecond laser-assisted cataract surgery (FLACS) versus phacoemulsification conventional surgery (PCS). *Methods*: Macular Optical Coherence Tomography OCT was analyzed in 42 patients preoperatively, 1 day, 12 days, 4 weeks and 6 weeks postoperatively according to the 9-field Early Treatment Diabetic Retinopathy Study (ETDRS) grid. Clinical findings were collected in both the FLACS group and the PCS group. *Results*: There was no significant difference in macular thickness between the FLACS and PCS groups (*p* > 0.05). However, from postoperative day 12 onwards, there was a significant increase in macular thickness observed in both groups (*p* < 0.001). In the FLACS group, a significant increase in visual acuity was observed on the first postoperative day, as compared to the PCS group (*p* = 0.006). *Conclusions*: The use of a low-energy high-frequency femtosecond laser has potentially no effect on postoperative macular thickness. In the FLACS group, visual rehabilitation was significantly faster as compared to the PCS group. No complications occurred intraoperatively in either group.

## 1. Introduction

Cataract is an age-related disease that affects millions of people worldwide every year. Left untreated, cataract will eventually lead to blindness. Cataract is in fact one of the most common causes of blindness in adults worldwide, with a significant negative impact on their quality of life [1]. The only effective treatment known so far is to perform a cataract surgery. Currently, most of those interventions are performed using conventional methods, i.e., by means of phacoemulsification [2]. Cataract surgery is one of the most frequently performed surgeries worldwide. With the ever-increasing average life expectancy and higher cataract prevalence, the number of surgeries required will increase in the coming years. The rehabilitation of visual acuity after surgery is of great importance. Therefore, surgical techniques have been subject to continuous refinement and improvements in order to the keep recovery time as short as possible. One of these refinements is the use of a femtosecond laser. It is, however, crucial to assess the potential impact of femtosecond laser technology on relevant tissue reactions, in particular at the level of the retina, and compare them with those seen for conventional surgical methods.

The dose of ultrasound energy delivered during the phacoemulsification step is relatively high as compared to the laser energy output delivered using a femtosecond laser, which may have an overall negative impact on neighboring tissues. It has been shown that phacoemulsification may alter macular structure and, consequently, negatively affect final surgical outcomes [3,4]. Over the years, femtosecond laser-assisted cataract surgery (FLACS) has gained more and more attention due to its potential advantages over the conventional phacoemulsification method. These include higher accuracy and reproducibility [5], with potentially better refractive outcomes [6], less endothelial cell loss [7,8,9], shorter effective phacoemulsification time [5,10] and supposedly lower intraoperative complication rates [11]. It is known that with FLACS, the phacoemulsification time can potentially be shortened and the applied energy can be reduced as a result. However, with FLACS, additional laser energy is applied to the tissue, which must also be taken into account, even though in our study the energy level is in the nanojoule (nJ) range.

In light of the above comparison between conventional cataract surgery and FLACS, the aim of this study was to assess both functional and anatomical effects of both of those surgical methods on the macula.

## 2. Materials and Methods

**Figure 1:**
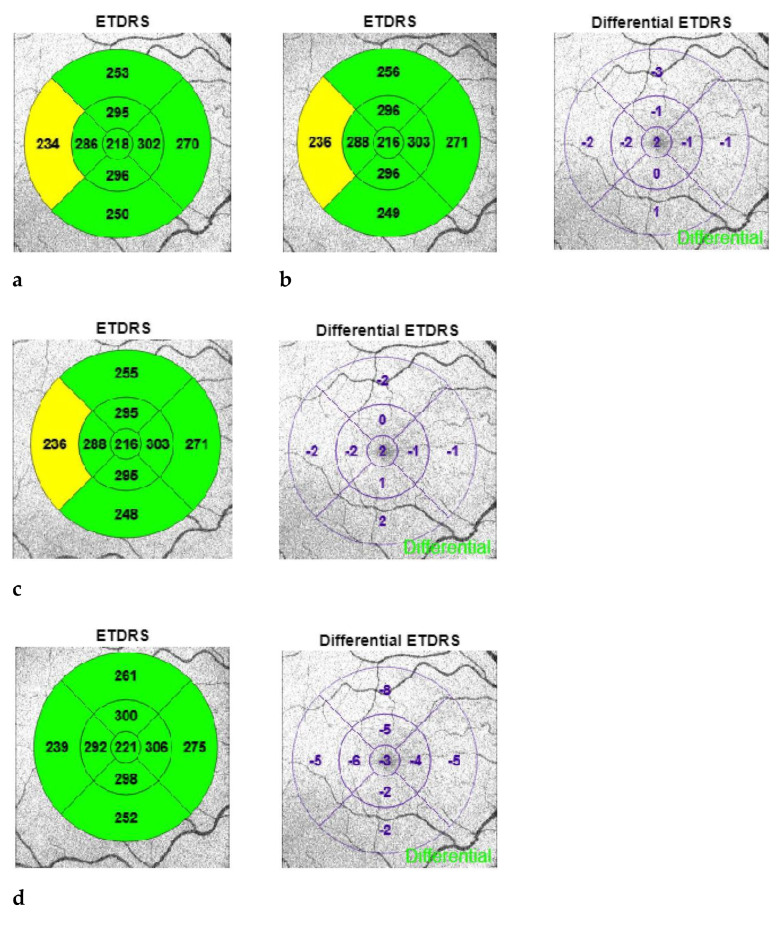

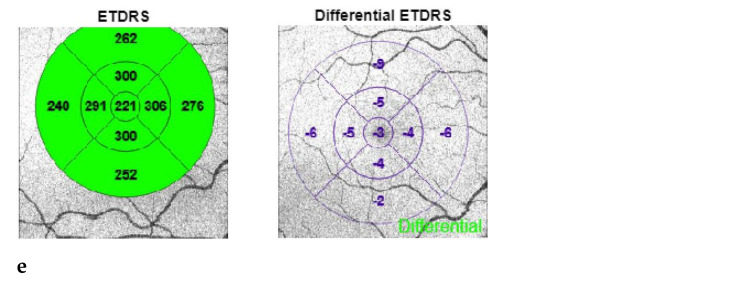
(**a**): preoperative, (**b**): 1 day postoperative, (**c**): 12 days postoperative, (**d**): 4 weeks postoperative, (**e**): 6 weeks postoperative.

In this retrospective study, we analyzed the OCT data of 42 patients after cataract surgery. For this study, only patients with age-related cataracts without concomitant ophthalmological diseases were included. The macular thickness was measured and analyzed according to the ETDRS (Early Treatment Diabetic Retinopathy Study) grid. We divided patients into 2 groups: one that underwent conventional cataract surgery, i.e., with phacoemulsification alone, and one that received femtosecond laser-assisted cataract surgery (FLACS). All procedures were performed by the same experienced surgeon (BP). Patients included in the study were treated within the time period from 1 February 2020 to 30 June 2022. For patients who underwent surgery on both eyes during that period, only one eye was included in the data analysis. The eye to be examined was randomly selected. A high-frequency femtosecond laser FEMTO LDV Z8 (Ziemer Ophthalmic Systems, Port, Switzerland) was used to perform the FLACS. This femtosecond laser has an energy output in the nanojoule (nJ) range. In the FLACS method, the eye is fixed with a suction ring with a target vacuum of 400 mbar. A balanced salt solution (BSS) is then placed in the liquid interface before the laser handpiece is docked onto the suction ring. The imaging technique of the femtosecond laser used is Spectral Domain OCT. OCT images can be used to precisely visualize all boundary surfaces of the anterior eye segments, such as the corneal structures of the anterior and posterior surfaces, the iris, the pupil and the pupil surfaces of the lens. An important advantage of OCT is the automatic three-dimensional recording of the anatomical structures with the additional inclusion of a safety distance. The femtosecond laser starts with an anterior capsulotomy with a target diameter of 5 mm. This is followed by lens fragmentation with six radial pie pieces, where a concentric circle is also cut perpendicular to these radial cuts. At the end of the femtosecond laser application, a main incision of 2.2 mm is cut with two additional paracenteses, which have a diameter of 0.8 mm. The pre-cut lens parts can then be easily removed via phacoemulsification, especially the cortical remnants using the irrigation/aspiration system (I/A). At the end of the operation, an intraocular lens is implanted in the capsular bag. For phacoemulsification, a Catharex 3 (Oertli Instrumente AG, Berneck, Switzerland) device was used in both groups. The following settings were applied: a 600 mmHg vacuum, a flow of 60 mL/min and an infusion height of 110 cm. The phacoemulsification device has an easyPhaco^®^ tip that enables gentle emulsification by applying a focused delivery of ultrasound energy in the axial axis. Using a lance, two 0.8 mm paracenteses are placed before a manual continuous capsulorhexis is formed with a cystotome, where the target diameter is 5 mm. The main incision of 2.2 mm is then created using a lance. After hydrodissection is performed, the stop-and-chop technique is used as the surgical method for removing the cataract. The cortex remnants are removed by means of I/A with consecutive IOL implantation in the capsular bag. The same viscoelastic (Pe-th-Visco 2.0% 2 mL) (ALBOMED GmbH, Germany) was used in both study groups. In addition to the ophthalmological status assessed as a function of the best corrected distance visual acuity (CDVA) expressed in log MAR notation, intraoperative parameters such as phaco time, effective phaco time (EPT), operation time and any complications were considered for the analysis. The severity of the cataract was graded based on the Lens Opacity Classification System (LOCS 3) [12]. The wide-angle OCT measurement (Topcon Triton Plus, Japan) of the macula was performed using a 9-field segmentation according to the ETDRS grid. This examination was carried out at each point of the postoperative follow-up and analyzed as shown using a patient example in Figure 1a–e.

A Scheimpflug topography (Galilei Topography, Ziemer Ophthalmic Systems, Switzerland) was also measured at each time point. In addition to the preoperative examination, postoperative examinations were performed on the 1st and 12th days, as well as at 4 and 6 weeks. The primary study objective was to investigate the behavior of macular thickness following cataract surgery performed with or without a second energy sources, namely femtosecond laser in addition to ultrasound (phacoemulsification). The secondary objective of the study was to evaluate the clinical outcomes related to the aforementioned cataract surgery methods. The protocol (2022-01363) of the study was approved by the Ethics Committee Nordwest-und Zentralschweiz (EKNZ, Basel, Switzerland).

All patients received Tobradex and Nevanac 0.1% drops (Alcon Laboratories, Inc., Fort Worth, TX, USA) three times daily for 4 weeks postoperatively. In addition, hyaluronic acid 0.15% drops (Thea Pharma, Clermont-Ferrand, France) were given for better lubrication of the ocular surface for the entire study period.

The IBM SPSS Statistics version 22.0 (IBM Corp., Armonk, NY, USA) software was used for statistical analysis purpose. For the power calculation, the Probability of Type I Error (alpha) = 0.001 was assumed. The reference proportion was set at 0.001. This resulted in a simple size of 39. The Kolmogorov–Smirnov and Shapiro–Wilk tests were used to test for normal distribution. Data sets were considered normally distributed if *p* > 0.05. For parametric data sets, the Pearson normality test, paired T-test and ANOVA test were used. The Friedmann test was applied to non-parametric data sets.

## 3. Results

A total of 42 eyes of 42 patients were included in this study. Of these, 21 eyes were included in the FLACS group, and 21 eyes belonged to the conventional group. The mean age in the FLACS group was 67 ± 15.8 years (range 50–95 years), and in the conventional group, it was 69 ± 12.6 years (range 53–90 years). No significant difference was found between the groups regarding participants’ age. There were 23 male (54.8%) and 19 female patients (45.2%). In the consecutive cases analyzed, the mean cataract grade assessed according to the LOCS3 classification was 3.5 ± 0.71 in the FLACS group and 2.3 ± 0.75 in the PCS group. Significance was found between the groups in terms of preoperative cataract grade (*p* < 0.001). Out of the 42 eyes that underwent cataract surgeries, 24 were left eyes and 18 were right eyes. There were no intraoperative complications observed.

The primary outcome was to compare the difference in macular thickness and morphology perioperatively between the FLACS group and the conventional (PCS) group. Macular thickness was assessed using the 9-field ETDRS scheme for each segment preoperatively, on day 1 and day 12 and at 4 and 6 weeks after cataract surgery.

**Figure 2:**
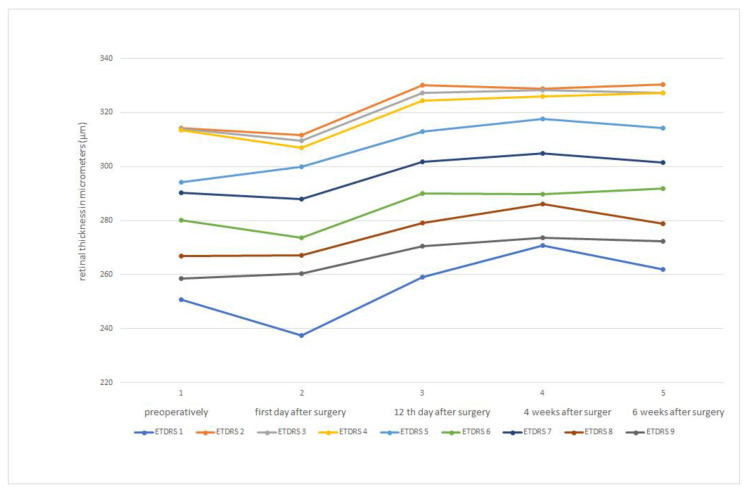
Shows a change in retinal thickness measured by ETDRS schema over the time course of the follow-up in the femtosecond laser-assisted cataract surgery. The *X*-axis represents time course where: 1 = preoperatively; 2 = 1st day after surgery; 3 = 12th day after surgery; 4 = 4 weeks after surgery; 5 = 6 weeks after surgery. The *Y*-axis represents retinal thickness measures in micrometers (μm).

**Figure 3:**
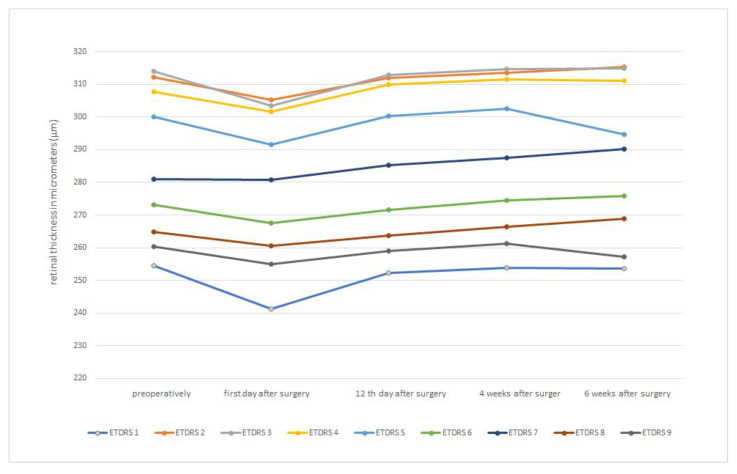
Shows a change in retinal thickness measured by ETDRS schema over the time course of the follow-up in the conventional ultrasound phacoemulsification cataract surgery. The *X*-axis represents time course where: 1 = preoperatively; 2 = 1st day after surgery; 3 = 12th day after surgery; 4 = 4 weeks after surgery; 5 = 6 weeks after surgery. The *Y*-axis represents retinal thickness measures in micrometers (μm).

Both the FLACS group and the PCS group had the same baseline macula thickness. In both groups, there was a reduction in macula thickness on postoperative day 1. Subsequently, both groups experienced an increase in macular thickness on postoperative day 12, as well as at 4 and 6 weeks after surgery. In the FLACS group, macular thickness flattened 6 weeks after surgery (Figure 2). A similar picture can be seen in the PCS group, where the macula thickness also flattens after 6 weeks (Figure 3). Table 1 shows the macula thicknesses of both groups, broken down by ETDRS 1–9 areas.

**Figure 4:**
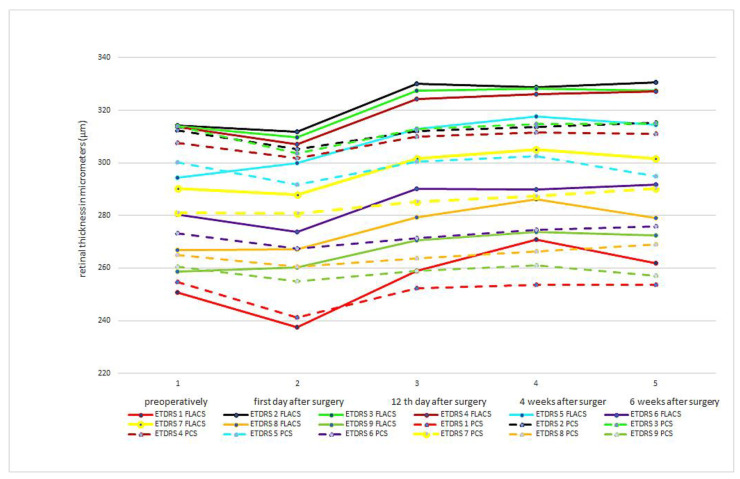
Shows the change in retinal thickness, measured according to the ETDRS scheme, over time during the follow-up of femtosecond laser-assisted cataract surgery and conventional phaco cataract surgery. The curves of the two groups were superimposed. The *X*-axis represents the time course, where: 1 = preoperative; 2 = 1st day after surgery; 3 = 12th day after surgery; 4 = 4 weeks after surgery; 5 = 6 weeks after surgery. The *Y*-axis shows the retinal thickness in micrometers (μm).

In Figure 4, we superimposed curves of the macular thickness values of the FLACS and PCS groups, obtained according to the ETRDS scheme, as a direct comparison at each pre- and postoperative time point. As in Figure 2 and Figure 3, the macular thickness reduction on the first postoperative day with a consecutive increase in macular thickness at the following time points can be seen clearly.

There was no significant difference in mean values of macular thickness between the FLACS and PCS groups at any time point, and the significance factor was *p* > 0.05. On the first postoperative day, macular thickness was thinner in all ETDRS areas in both groups compared to the preoperative measurement. However, the difference was not significant (*p* = 0.40 for the FLACS group and *p* = 0.26 for the PCS group, respectively). However, a significant increase in macular thickness at 12 days, 4 weeks and 6 weeks postoperatively, with *p* < 0.001 for both groups compared to the preoperative values for all ETDRS areas, was observed.

The cataract grade was 3.5 ± 0.71 in the FLACS group and 2.3 ± 0.75 in the PCS group. The cataract density was significantly higher in the FLACS group than in the PCS group, with *p* < 0.001. The phacoemulsification time was 1.2 ± 1.25 s in the FLACS group and 2.2 ± 2.81 s in the PCS group, which is significantly shorter in the FLACS group *p* = 0.009. It was found that the Effective Phaco Time (EPT) was 0.8 ± 0.94 s in the FLACS group and 1.8 ± 1.79 s in the PCS group, which was also significantly shorter in the FLACS group (*p* = 0.006).

**Figure 5:**
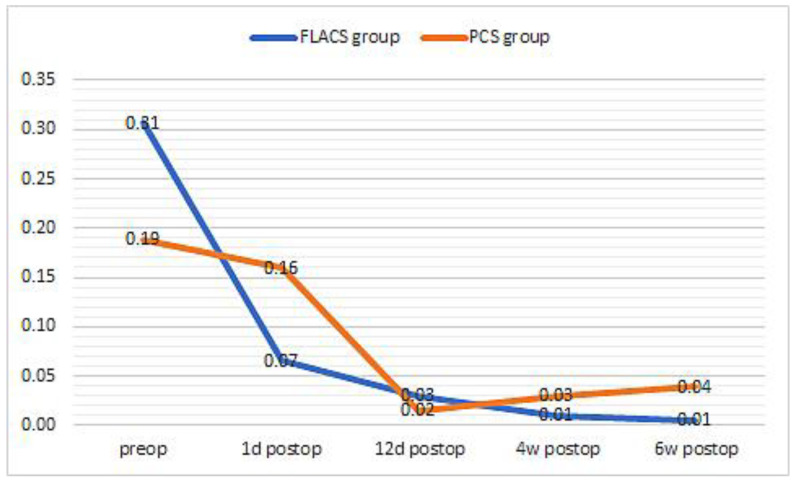
Visual acuity in the FLACS and PCS group over time.

Preoperatively, the BCVA logMAR was 0.31 ± 0.267 in the FLACS group and 0.19 ± 0.090 in the PCS group. There was no significant difference between the groups (*p* > 0.05). On the first postoperative day, there was a significant increase in visual acuity in the FLACS group to 0. 07 ± 0.067 logMAR *p* = 0.006 in the FLACS group and 0.16 ± 0.099 logMAR in the PCS group, which was not significant *p* = 0.43. The improvement in visual acuity on the first postoperative day was statistically significantly higher in the FLACS group than in the PCS group *p* < 0.001. At 12 days, 4 weeks and 6 weeks postoperatively, visual acuity in the FLACS group was 0.03 ± 0.073 logMAR, 0.01 ± 0.064 logMAR and 0.01 ± 0.039 logMar, respectively, which was statistically significant as compared to the preoperative baseline *p* < 0.001. At 12 days, 4 weeks and 6 weeks postoperatively, visual acuity in the PCS group was 0. 02 ± 0.049 logMAR, 0.03 ± 0.057 logMAR and 0.04 ± 0.070 logMar, which is also a significant improvement within each group compared to the preoperative values *p* < 0.001. From postoperative day 12 onwards, there was no significant difference between the two groups *p* > 0.05 in terms of reported visual acuity values (Figure 5).

In the FLACS group, a mean corneal endothelial cell count of 2454 ± 282 cells/mm^2^ (range 1740–3121) was observed preoperatively. On day 1 and day 12 postoperatively, as well as at 4 and 6 weeks, the mean corneal endothelial cell count (CD) was 2523 ± 272 cells/mm^2^ (range 1805–2932), 2459 ± 328 cells/mm^2^ (range 1810–2956), 2405 ± 300 cells/mm^2^ (range 1871–2937) and 2368 ± 290 cells/mm^2^ (range 1795–2938). There was no statistically significant corneal endothelial cell count reduction in the FLACS group at any time point (*p* > 0.05).

In the PCS group, the preoperative mean corneal endothelial cell (CD) count was 2478 ± 386 cells/mm^2^ (range 1241–2994). On postoperative day 1, the mean corneal endothelial cell count decreased to 2327 ± 444 cells/mm^2^ (range 1310–2859) which was significant with *p* = 0.016. On postoperative day 12, as well as after 4 and 6 weeks, the mean corneal endothelial cell count was 2305 ± 494 cells/mm^2^ (range 1356–3028), 2268 ± 482 cells/mm^2^ (range 1394–2949) and 2275 ± 477 cells/mm^2^ (range 1353–2968). From postoperative day 12, there was no further statistically significant corneal endothelial cell count reduction with *p* > 0.05.

There was no statistical difference in corneal endothelial cell count between the FLACS and PCS groups at baseline preoperatively (*p* = 0.828). On postoperative day 1, there was a significant difference in corneal endothelial cell count between the groups with *p* = 0.05. On postoperative day 12, the significant difference between the groups increased slightly (*p* = 0.04), as well as at 4 weeks postoperatively (*p* = 0.021), and then decreased again slightly at 6 weeks postoperatively (*p* = 0.03).

## 4. Discussion

In our study, we observed a non-significant decrease in the macular thickness on the first postoperative day, whilst there was a significant increase in macular thickness observed at 12 days, 4 weeks and 6 weeks. However, it should be noted that there was no significant difference between the FLACS and the PCS groups. These macular thickness dynamics did not seem to have an impact on clinical relevance regarding visual acuity at any analyzed time point. What is remarkable in our study is the observation that, on the one hand, the cataract grade was significantly higher in the FLACS group than in the PCS group, but on the other hand, the EPT was significantly lower in the FLACS group than in the PCS group, despite the higher cataract grade. At the same time, with FLACS, a shorter EPT was observed despite the higher lens density, which subsequently led to significantly faster visual rehabilitation, as compared to PCS.

In another study, macular thickness was investigated in myopic patients after cataract surgery using only the PCS surgical technique. Although postoperative thickening of the macula was observed, it was not significant [13]. A follow-up study sought to compare FLACS with PCS. In this study, a conventional femtosecond laser (Centurion Vision System, Alcon, Fort Worth, TX, USA) was used, which has an energy output in the range of µJ, i.e., much higher than the femtosecond laser used in the current study with the range of nJ. On the one hand, the authors observed that less phaco time and, thus, phaco energy was used in the FLACS group, and on the other hand, the femtosecond laser energy was delivered, which logically does not exist in the PCS. Despite this initial situation, no significant differences in macular thickness were found between the FLACS group and the PCS group [14]. This is comparable to our study data, although we applied far less femtosecond energy, which prompts the conclusion that low-pulse laser energy aspect seems to provide a minor contribution to overall outcomes related to postoperative macular thickness. Another study confirmed our results, but this work has a limitation and is therefore not directly comparable as five surgeons were involved in performing surgical procedures. Nevertheless, no significant difference in postoperative macular thickness was found between the FLACS group and the PCS group [15]. Another study also analyzed macular thickness using the ETDRS macular classification following PCS patients [16]. However, this study was conducted exclusively with the PCS technique. Decreases in thickness in most retinal areas were observed in the ETDRS subfield in the central 1 mm circle and 1–3 mm circles encompassing the superior, temporal, inferior and nasal subfields on the first postoperative day. Significant macular thickness was observed at all further postoperative examinations and did not return to the preoperative baseline at the final examination. This is quite comparable to the macular thickness values observed in our study. Interestingly, and different from our work, the EPT and total energy application correlated with macular thickness measurements. Apparently, this larger phaco energy source seems to have a clinical relevance on macular thickness as well [16]. The slight non-significant decrease in macular thickness on the first postoperative day, as we also observed, is due potentially to the systemic application of acetazolamide [17,18]. The correlation between phaco energy and the significant increase in macular thickness was also confirmed in yet another study [19]. In general, the FLACS method is described to have specific advantages over PCS, such as faster visual rehabilitation, which is confirmed by our study. It is also considered to be gentler overall, as shown in various other publications [5,6,7,8,9,10,11]. In our study, FLACS seems to have no additional advantage over the PCS method as a function of macular thickness, but it also has no disadvantage. In two other studies that used the same femtosecond laser for the study as we did, no correlation between laser application and macular thickness was found either [20,21]. This is an important finding that low-energy high-frequency femtosecond lasers, as we used in the study, seem to have no effect on the macular thickness. It is possible that the energy level is not sufficient to cause a side effect on that particular structure. However, it is conceivable that a much higher energy application of the femtosecond laser could cause potential alterations in tissue such as the macula. Nevertheless, according to the literature, it seems to be undisputed that phaco energy correlates very well with postoperative macular thickness. However, we could not show this in our work. The reason is probably because although the EPT is significantly shorter in the FLACS group with 1.2 ± 1.25 s, the EPT in the PCS group is also objectively very short with 2.2 ± 2.81 s. If the difference between the EPT was greater, one might also potentially expect to see a correlation between macular thickness and delivered phaco energy. In addition to the impact of phaco energy on macular thickening, other factors also play a role in the morphological changes of the macula, such as inflammatory parameters, and might be responsible for the different outcome.

Another point that correlates very well with the shorter EPT in the FLACS group compared to the PCS group is the corneal endothelial cell pattern. In our study, we found that we had significantly less corneal endothelial cell reduction in the FLACS group at each measured time point compared to the PCS group. Our observation compares very well with another study that also used a low-energy high-frequency femtosecond laser [9].

In our study, there was a significant increase in visual acuity on the first postoperative day in the FLACS group, as compared to the PCS group. No significant difference was found at any of the later time points. In this sense, there was a faster visual rehabilitation in the FLACS group, which was also found in other studies [22,23]. The faster recovery of visual acuity in the first postoperative period with FLACS compared to PCS can be very important in some patient groups. However, in other studies, no significant difference in postoperative visual acuity was found between the two groups [24,25,26]. The reason may be that these studies used conventional femtosecond lasers with much higher applied laser energy in the µJ range, as compared to the laser that we used. In addition, tissue bridges that often develop during the surgery have to be mechanically mobilized, which can lead to tissue swelling that can negatively influence visual acuity values.

## 5. Conclusions

No significant difference in final macular thickness was found between the FLACS group and the PCS group. Thus, there is much evidence to suggest that the high-frequency low-energy femtosecond laser has no effect on macular thickness. The FLACS group experienced a faster recovery of visual acuity compared to the PCS group, showing significant improvement in visual acuity as soon as day 1 postoperatively.

## Figures and Tables

**Table 1:** Macula thickness in the FLACS and PCS groups.

Flacs in µm	ETDRS1	ETDRS2	ETDRS3	ETDRS4	ETDRS5	ETDRS6	ETDRS7	ETDRS8	ETDRS9
preoperative	250 ± 60	314 ± 48	314 ± 34	313 ± 39	294 ± 56	280 ± 33	290 ± 30	266 ± 35	258 ± 41
1d postoperative	237 ± 40	311 ± 28	309 ± 34	306 ± 26	299 ± 22	273 ± 19	287 ± 26	267 ± 25	260 ± 20
12d postoperative	259 ± 59	330 ± 50	327 ± 35	324 ± 37	312 ± 36	290 ± 47	301 ± 32	279 ± 32	270 ± 29
4w postoperative	270 ± 60	328 ± 53	328 ± 35	326 ± 46	317 ± 50	289 ± 45	305 ± 31	286 ± 36	273 ± 36
6w postoperative	261 ± 59	330 ± 48	327 ± 32	327 ± 38	314 ± 38	291 ± 46	301 ± 30	278 ± 29	272 ± 29
PCS in µm									
preoperative	254 ± 15	312 ± 27	313 ± 26	307 ± 27	300 ± 23	273 ± 23	280 ± 27	264 ± 23	260 ± 24
1d postoperative	242 ± 30	305 ± 25	305 ± 28	303 ± 25	292 ± 22	267 ± 23	281 ± 24	261 ± 21	254 ± 22
12d postoperative	252 ± 19	311 ± 26	312 ± 26	309 ± 25	300 ± 23	271 ± 22	285 ± 22	263 ± 20	259 ± 22
4w postoperative	253 ± 18	313 ± 25	314 ± 25	311 ± 25	302 ± 23	274 ± 23	287 ± 24	266 ± 22	261 ± 22
6w postoperative	253 ± 37	315 ± 25	315 ± 27	311 ± 28	294 ± 38	275 ± 22	290 ± 24	268 ± 20	257 ± 32

## Data Availability

The data presented in this study are available on request from the first and the corresponding authors; in particular, the data sets are archived in the clinics treated. The data are not publicly available as they contain information that could compromise the privacy of the participants.
